# Reporter Genes for Brain Imaging Using MRI, SPECT and PET

**DOI:** 10.3390/ijms23158443

**Published:** 2022-07-30

**Authors:** Tianxin Gao, Pei Wang, Teng Gong, Ying Zhou, Ancong Wang, Xiaoying Tang, Xiaolei Song, Yingwei Fan

**Affiliations:** 1 School of Life Science, Beijing Institute of Technology, Beijing 100081, China; gtx@bit.edu.cn (T.G.); wpei02288@sina.com (P.W.); zhouying2587@gmail.com (Y.Z.); awang@bit.edu.cn (A.W.); xiaoying@bit.edu.cn (X.T.); 2 Center for Biomedical Imaging Research, School of Medicine, Tsinghua University, Beijing 100084, China; gongt923@163.com; 3 School of Medical Technology, Beijing Institute of Technology, Beijing 100081, China

**Keywords:** reporter gene, MRI, radionuclide imaging, brain imaging

## Abstract

The use of molecular imaging technologies for brain imaging can not only play an important supporting role in disease diagnosis and treatment but can also be used to deeply study brain functions. Recently, with the support of reporter gene technology, optical imaging has achieved a breakthrough in brain function studies at the molecular level. Reporter gene technology based on traditional clinical imaging modalities is also expanding. By benefiting from the deeper imaging depths and wider imaging ranges now possible, these methods have led to breakthroughs in preclinical and clinical research. This article focuses on the applications of magnetic resonance imaging (MRI), single-photon emission computed tomography (SPECT), and positron emission tomography (PET) reporter gene technologies for use in brain imaging. The tracking of cell therapies and gene therapies is the most successful and widely used application of these techniques. Meanwhile, breakthroughs have been achieved in the research and development of reporter genes and their imaging probe pairs with respect to brain function research. This paper introduces the imaging principles and classifications of the reporter gene technologies of these imaging modalities, lists the relevant brain imaging applications, reviews their characteristics, and discusses the opportunities and challenges faced by clinical imaging modalities based on reporter gene technology. The conclusion is provided in the last section.

## 1. Introduction

Molecular imaging is an imaging technique that visualizes, characterizes, and measures biological processes in vivo at the molecular and cellular levels [1]. Reporter gene imaging is a critical technical route for molecular imaging, which introduces or expresses imaging agents into cells through so-called reporter genes. Reporter genes are those genes that, when introduced into target cells (e.g., brain tissues, cancer, and circulating white cells), produce a protein receptor or enzyme that binds, transports, or traps a subsequently injected imaging probe, which becomes the contrast agent for reporter gene imaging [2]. Reporter gene imaging is developing very rapidly for monitoring cell therapy and gene therapy by providing critical information on the biodistributions, magnitudes, and durations of viral gene expressions. Imaging the brains of large animals or humans on a large scale has become the next challenge of reporter gene imaging.

Among multiple imaging modalities, fluorescence reporter genes have drawn great attention; however, penetration depths limit their in vivo application [3]. Recently, other imaging modalities, including magnetic resonance imaging (MRI), single-photon emission computed tomography (SPECT), positron emission tomography (PET), and ultrasound (US) imaging, have been explored in the field of reporter genes [2]. The advantages of genetically encoded fluorescent imaging are high spatiotemporal resolution, high sensitivity, and high molecular specificity, while some conventional imaging modalities that use modern reporter gene indicators are effective in clearly examining the brains of larger subjects in deep organs and on large scales. Compared with optical reporter gene imaging, other types of reporter gene imaging have a variety of properties, as shown in Table 1. For the imaging depths and scales, clinical imaging techniques provide better performance, while fluorescence reporters are excellent in terms of their temporal–spatial resolution, noninvasiveness, molecular and cell specificity, and sensitivity.

Brain imaging includes imaging of diseases, such as neurodegenerative disease and glioma, and foundational brain research, which can be divided into two categories: macroscopic, noninvasive human cognitive neuroscience and invasive reductionist neurobiology [4]. Reporter gene base brain imaging includes both of these categories. Nonoptical imaging modalities are commonly used in clinics, which means that such studies involve direct human applications.

Observing brain activity is a dream of researchers. Gene-encoded fluorescent indicators that are used to dynamically monitor neurotransmitters and neuromodulators with fluorescence imaging have recently achieved great breakthroughs and have been reviewed in excellent studies [3]. We note that, for clinical imaging modalities, such as MRI, radionuclide imaging, and US, some breakthroughs have been achieved in reporter gene-based brain studies. A number of positive reviews have summarized reporter gene materials [5], the history of MRI in brain activity detection [4], transgene-based strategies in nuclear-based imaging [6], nanoparticle-mediated brain imaging [7], and reporter gene imaging in oncolytic virotherapy and gene therapy [2]. Here, we focus on the recently developed nonoptical techniques of reporter gene imaging used in brain imaging.

## 2. Principle of Reporter Gene Imaging

The two basic elements of reporter gene imaging consist of reporter genes and their respective imaging probes (which are also the referred to as imaging agents, substrates, or imaging reporters in other references). The accumulation of imaging probes directly depends on the protein products of reporter gene expression, thereby imaging the reporter gene. Because it only monitors living cells, it can accurately provide important information such as that of survival, proliferation, migration, differentiation, and functional integration of transplanted cells in vivo [1,8,9].

### 2.1. Reporter Gene Imaging with MRI

MRI employs the resonance properties of atomic nuclei subjected to strong magnetic fields and radiofrequency pulses to generate signals and reconstruct images. MRI reporter genes can directly or indirectly produce magnetic resonance contrast signals that are based on the expressions of coding enzymes, receptors, metalloproteins, etc., which can specifically combine with MRI contrast agent [10,11,12,13]. MRI reporter genes can be used to longitudinally monitor the cell migration process and gene expressions by using noninvasive imaging [14]. The main types of existing MRI reporter genes include reporter genes that encode enzymes (e.g., tyrosinase), reporter genes that encode receptors on cells (e.g., transferrin receptor (TfR)), endogenous reporter genes (e.g., ferritin and aquaporin 1 (AQP1)), and reporter genes that express CEST-detectable proteins (e.g., lysine rich-protein (LRP)) [15].

**Figure 1:**
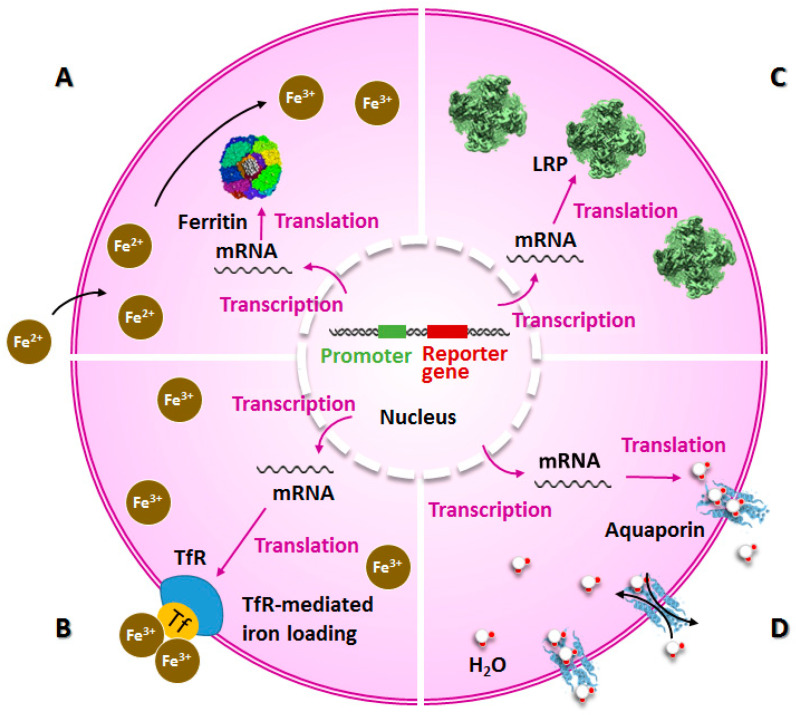
The principle of MRI reporter genes commonly used in brain imaging. (**A**) The expression of ferritin will cause intracellular iron particles to aggregate, resulting in a decrease in the T2 signal in MRI images. (**B**) The expression of TfR increases the iron uptake of cells and shows low signal intensity on T2 images of MRI. (**C**) LRP has a high chemical exchange rate and can be used in CEST MRI. (**D**) Aquaporin can increase water diffusivity and produce contrast in diffusion-weighted MRI.

As shown in Figure 1A,B, the most classic contrast agent for MRI reporter genes is iron. As a ubiquitous protein in the cells of various organisms, ferritin is assembled by two subunits, a heavy chain and a light chain. The proportions of light and heavy chains vary in different tissues. The ferritin heavy chain (FTH1) contains ferrous oxidase and can combine with iron oxide to transform unstable Fe^2+^ into stable, insoluble, and nontoxic Fe^3+^ forms. The light chain mainly increases the activity of FTH1 and stabilizes ferritin. Ferritin can specifically bind to iron, which results in accumulations of intracellular iron particles and decreases in T2 signals [16], and it is one of the most commonly used reporter genes in MRI (Figure 1A). The transferrin receptor (TfR) is another commonly used MRI reporter gene. TfR can bind to transferrin and transfer iron into cells via endocytosis, thus reducing the T2 relaxation time (Figure 1B) [14,17]. Lysine-rich protein (LRP) is an artificially designed gene. Due to the uniquely high chemical exchange rate of poly-l-lysine, LRP can be used as a reporter gene in chemical exchange saturation transfer (CEST) MRI (Figure 1C) [18,19]. The CEST mechanism occurs because exchangeable protons have chemical shifts that are different from water. These protons are selectively saturated and exchanged with water molecules, thus reducing the water signal. Magnetic resonance diffusion-weighted imaging (DWI) is an imaging method that uses MRI to observe the microdiffusion movements of water molecules in living tissues. It can noninvasively image the structures and physiological functions of living brain tissues. The apparent diffusion coefficient (ADC) is used to describe the diffusion rate of water molecules in DWI. A positive correlation exists between them. When water molecules with freer diffusion are dephasing, the level of signal loss is greater, the signal is weaker, and it appears darker in DWI, and vice versa [20,21]. Aquaporins mediate the selective exchange of water-conducting molecules across plasma membranes in many cell types, and their expressions are related to water diffusivity and DWI signals in several disease states [22,23]. Previous studies have shown that overexpression of aquaporin can increase tissue water diffusivity without affecting viability, and contrasts are observed in diffusion-weighted MRI (Figure 1D) [24].

### 2.2. Reporter Gene Imaging with Radionuclides

Radionuclide imaging refers to SPECT and PET. SPECT detects gamma rays that are produced by the decay of the radioactive isotopes used in imaging, and it has been developed to elucidate the basic molecular neurodegeneration mechanism in PD, AD, and drug addiction, as well as to improve therapeutic strategies with minimum adverse effects. PET uses the annihilation of positrons (emitted by decaying radioisotopes in the imaging agent) and electrons to generate 510 keV collinear photons, which are detected simultaneously to generate a three-dimensional map of radioactivity distributions in the body [6]. Radionuclide imaging has very high sensitivity and good penetration ability in tissues, and it can be used in clinical practice. It has been widely used for noninvasive tracing and monitoring of living cells.

**Figure 2:**
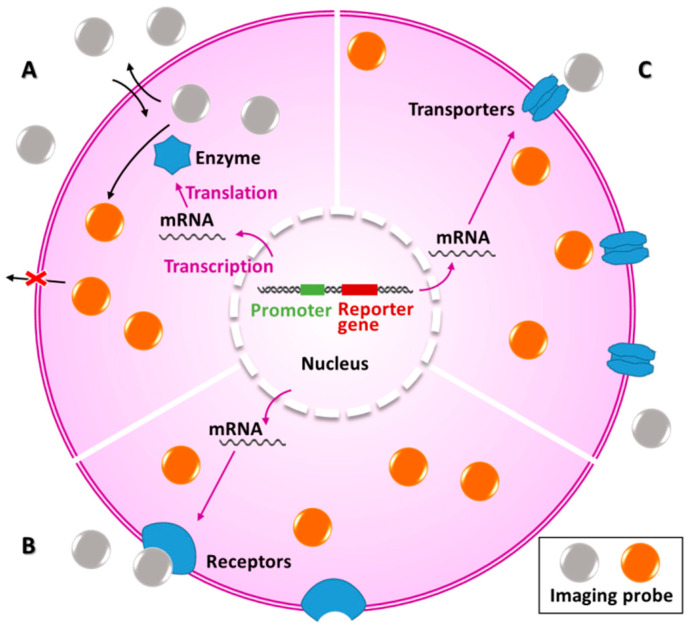
The major types of reporter gene expression in radionuclide imaging, based on (**A**) enzymes, (**B**) receptors, and (**C**) transporters.

PET imaging of reporter gene expression utilizes reporter gene imaging agents that are labeled by positron radionuclides. Currently, there are three types of commonly used radionuclide reporter gene imaging systems based on enzymes, receptors, and transporters, as shown in Figure 2 [6,25]. The most common reporter genes of enzymes are herpes simplex virus 1 thymine kinase (HSV1-tk) [9] and human Δ-mitochondrial thymine kinase type 2 (hΔtk2) [26]. The most common reporter genes are human somatostatin receptor type 2 (hSSTR2) [27] and dopamine D2 receptor (D2R) [28]. The most common reporter genes of transporters are human sodium–iodide symporter (hNIS) [29] and human norepinephrine transporter (hNET) [2,30].

One of the first and hence most intensively studied reporter genes, HSV1-tk, is also a suicide gene that adds an extra layer of control to ensure safety. NIS imaging is the most mature reporter gene imaging method used in human clinical trials and is more sensitive and longer lasting than HSV1-tk.

The SPECT imaging system was developed to elucidate the basic molecular neurodegeneration mechanism in PD, AD, and drug addiction, as well as to improve therapeutic strategies with minimum amounts of adverse effects. PET imaging can provide diagnosis and treatment guidance for tumors and cardiovascular and brain diseases. PET imaging of reporter gene expressions is capable of monitoring gene and cell therapy [6]. In brain studies, brain cancer and neurodegenerative disorders are the major diseases diagnosed and monitored by reporter gene expression PET imaging, as discussed thoroughly in Section 3.2.

## 3. Reporter Gene Imaging in Brain Studies

### 3.1. Brain Imaging of Reporter Genes with MRI

In view of the diversity, high resolution, and noninvasive nature of MRI, MRI imaging of the brain can be used in a variety of applications, such as observing the process of virus infection through in vivo imaging, longitudinally monitoring cell migration and proliferation during cell therapy, noninvasive detection of neural connections, and monitoring neurogenesis. The majority of studies of MRI reporter genes in brain imaging are listed in Table 2.

**Table 2:** Reporter genes in brain imaging of MRI.

Class	Reporter Gene	Imaging Mode	Properties	Ref.
Receptor	TfR–FTH	T2WI	Shows increased contrast on T2-weighted brain images.	[31]
Endogenous reporter genes	Ferritin–EGFP	T2WI, FI	Synaptically connected neural network can be detected by ex vivo MRI and fluorescence imaging.	[32]
	Ferritin	T2WI	Realizes the accumulation of iron ions, resulting in a change in MR signal in the infected regions. Allows in vivo MRI to observe the process of virus infection and detect the neural circuits of living animals.	[33]
	FTH–EGFP	T2WI, FI	Tracks the tropism and fate of MSCs after systematic transplantation into orthotopic gliomas	[34]
	FTH1	DWI, SWI, T2WI	Allows in vivo detection of BMSCs transplanted due to cerebral ischemia/reperfusion injury and to treat intervention. FTH1-BMSC transplantation in the treatment of focal cerebral infarction is safe, reliable, and traceable by MRI; SWI is more sensitive than T2WI.	[35]
	FerrH	T2WI	Allows noninvasive visualization of neurogenesis in normal and ischemic rat brains using T2-weighted MRI	[36]
	IFNβ–FTH	T2WI	Traces MSCs and detects the therapeutic effect of IFNβ on glioma.	[37]
	FTH1–iRFP–EGFP	T2WI, NIF, FI	Tracks cells transplanted into the brain of mice during cell therapy by multimodal imaging.	[38]
	AQP1	DWI	The expression of AQP1 can provide DWI image contrast, which makes it possible to image the gene expression of intracranial tumor xenografts.	[24]
	EGFP–AQP1	DWI	Detection of astrocytes by fluorescence imaging and diffusion-weighted MRI.	[39]
	AQP1	DWI	Detection of brain-wide neural networks in vivo by metal-free MRI.	[40]
CEST	LRP	CEST MRI	Increases the contrast of CEST images of cell lysates and rat gliomas.	[18]
	rd LRP	CEST MRI	The CEST MRI contrast of mouse brain tumor is higher than that of LRP.	[41]
	dNKs	CEST MRI	Accurate localization in mouse intracranial tumor model. Realizes noninvasive two-color imaging of polygenes in deep tissue of living animals.	[42]

**Figure 3:**
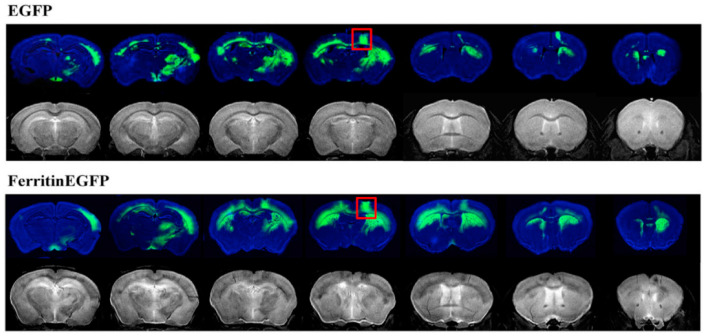
Brain MRI (lower in both groups) and fluorescence images (upper). Four days after rVSV- EGFP or rVSV–Ferritin–EGFP was injected into the SC (red box) of mice, the mice were killed, and the images were obtained. Reproduced with permission from Ref. [32], 2019, Elsevier.

**Figure 4:**
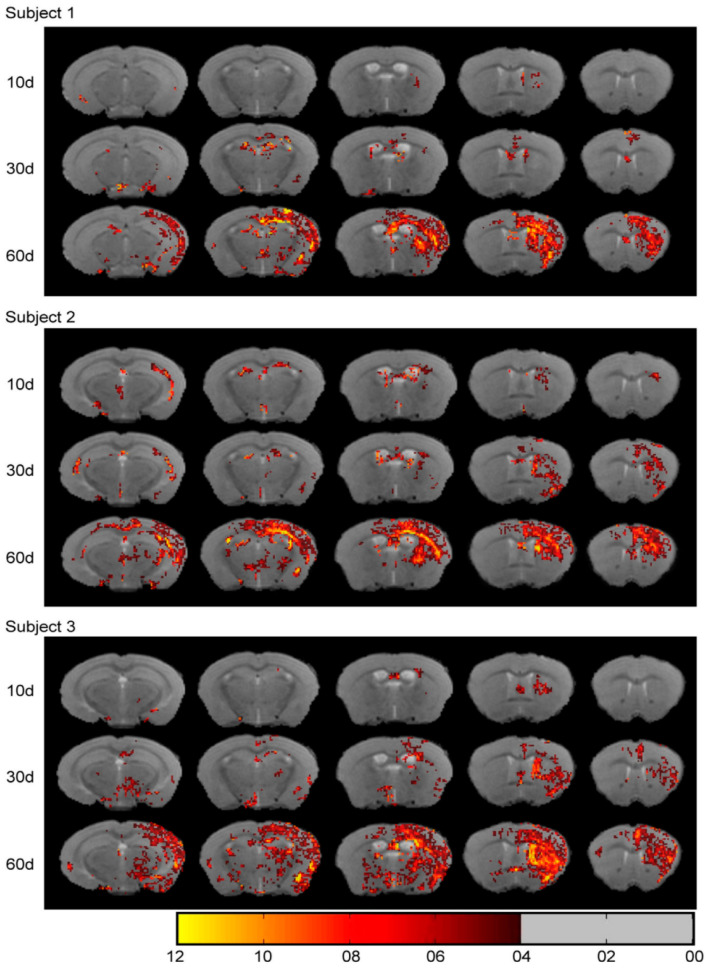
After different times (10 days, 30 days, or 60 days) of the rAAV2-retro–CAG–Ferritin injection, the CPU connection area is displayed by MRI in vivo (expressed by the change in T2 relaxation times). A color change (red–yellow) is used to indicate a change in T2 relaxation time (4–12 ms). Reproduced with permission from Ref. [33], 2021, Wiley.

Visualization of neural networks helps provide a better understanding of the mechanisms of some brain functions and brain diseases. In the study of Wang et al. [32], vesicular stomatitis virus (VSV), a neurovirus that can spread sequentially in synaptic networks, was used to carry chimeric genes that encode ferritin and enhanced green fluorescent protein (EGFP). After recombinant VSV (rVSV) was injected into the somatosensory cortex (SC) of mice, the structural nerve connections were detected by MRI and fluorescence imaging. However, due to the high toxicity of VSV, mice infected with rVSV cannot survive for long periods, and in vivo MRI research is not allowed. The MRI and fluorescence images obtained after the death of mice are shown in Figure 3. In another study conducted by the team [33], hypotoxicity virus adeno-associated virus (AAV) was used as a vector to integrate the ferritin coding gene to obtain a ferritin coding viral vector (e.g., rAAV2-retro–CAG–Ferritin), which was injected into the caudate putamen (CPu) of mice to achieve noninvasive detection of neural networks in vivo. The CPu connection area was displayed by MRI at different time points after rAAV2-retro–CAG–ferritin injection (Figure 4). The team then focused on describing the activity of astrocytes, which are a major component of the central nervous system. They used the EGFP–AQP1 fusion gene of EGFP and aquaporin 1 (AQP1) as the reporter gene, detected astrocytes by fluorescence imaging and diffusion-weighted MRI, and established a new technique for the noninvasive detection of astrocytes in vivo for the first time [39]. In the newly published work [40] of the team, a tool virus rAAV-retro–AQP1–EGFP expressing nonmetallic magnetic resonance reporter gene AQP1 was prepared and used for in vivo brain-wide neural network detection. Three weeks after microinjection of virus rAAV-retro–AQP1–EGFP into the CPU brain area of mice, the changes in magnetic resonance signals in multiple brain regions (CPU, Ctx, BLA, Ins, Tha, HIP, etc) were observed by diffusion-weighted MRI, and the rapid imaging of specific brain region-related brain networks was successfully realized (increase from 60 days [33] to 21 days). The project also combined with the Cre–loxP system to prepare a brain network expressing Cre-dependent AQP1-related tool virus rAAV-retro–DIO–AQP1–EGFP for in vivo detection of specific neuronal types in specific brain regions. This strategy provides a solid foundation for the visualization of neural networks in rodents and nonhuman primates.

Mesenchymal stem cells (MSCs) can cross the blood–brain barrier and tend to accumulate in tumors [43]; hence, they can be developed as cell carriers to treat gliomas [44,45,46]. Longitudinal in vivo monitoring of the migration and fate of MSCs is very important for the development of MSCs as cell carriers. Cao et al. [34] used a lentivirus as a vector to carry the ferritin heavy chain (FTH) and EGFP genes and transferred it to MSCs. MCSs expressing reporter genes were injected into a rat glioma model using different injection methods (e.g., arterial injection, intravenous injection, and stereotactic injection), and the homing and migration behaviors of MSCs were detected by MRI. The results showed that arterial injections of MSCs had a clear ability to treat glioma. MRI based on the ferritin reporter gene can be used to trace the tendency of MSCs to accumulate in glioma in vivo. Mao et al. [37] constructed MSCs with high expressions of interferon-β (IFNβ) and FTH in a similar manner. MRI was used to evaluate whether MSCs can be used as cell carriers to carry IFNβ to treat brain tumors, which provides a new option for treating brain tumors. Studies have shown that FTH-based MRI can monitor this treatment process.

**Figure 5:**
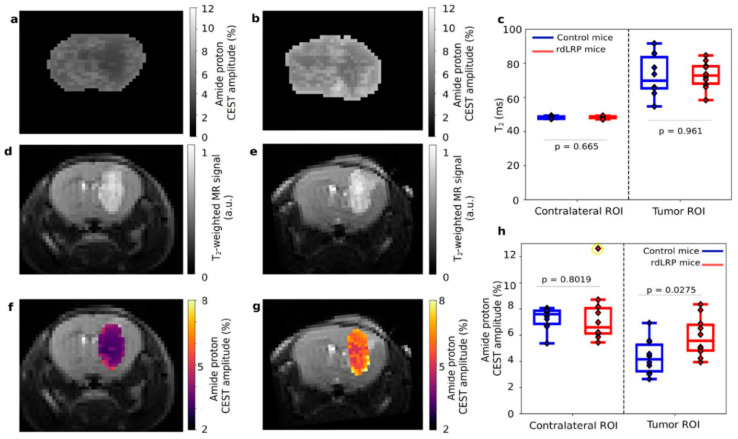
In vivo imaging of the rd LRP. (**a**,**b**) The amide proton CEST amplitudes of glioma cells that did not express rd LRP (**a**) and expressed rd LRP (**b**) in mouse brain. (**c**) Comparison of the T2 relaxation time of the two groups. The results showed no significant difference, and there was edema in the tumor area. (**d**,**e**) T2-weighted imaging corresponding to (**a**,**b**) respectively. (**f**,**g**) Overlay of the amide proton CEST contrast (obtained using a frequency selective saturation pulse (**a**,**b**)) on the (**d**,**e**) image. (**h**) Comparison of CEST amplitudes of amide proton. CEST signal increased significantly in rd LRP group. Reproduced with permission from Ref. [41], 2020.

MRI reporter genes are also used to study brain viral infections and for brain tumor imaging. Oncolytic viruses can be used to treat malignant tumors, such as glioblastoma [47,48]. The infection process is expected to be observed by MRI. In clinical trials, Christian et al. [18] integrated the lysine-rich protein (LRP) gene into a herpes simplex virus-derived oncolytic virus G47∆ virus. CEST MRI was used to detect gliomas in rats before and 8 h after injection of G47∆-LRP or a control G47∆-empty virus. The contrast increased in tumors of CEST images after infection with G47∆-LRP virus. This study shows that LRP can be used as a reporter gene for real-time monitoring of virus transmission, but the highly repeated gene sequence of LRP may lead to DNA recombination events and expression of a series of truncated LRP protein fragments, which limits the sensitivity of CEST imaging. To address this problem, Perlman et al. [41] redesigned an LRP reporter (rd LRP) without DNA repeat sequences and improved its CEST MRI contrast. The in vivo CEST MRI of brain tumors in mice is shown in Figure 5.

Additional innovative studies have been reported. Hyla et al. [42] developed a system called GeneREFORM, calculated and designed a group of two-color reporter genes and probes, and established a two-color gene imaging system. With the aid of existing MRI technology, GeneREFORM can accurately locate and achieve noninvasive two-color imaging of multiple genes in the deep tissues of living animals. The GeneREFORM system is also applicable to nontumor models.

There are also potential MRI reporter genes that can be used for brain imaging, notably gas vesicles (GVs). GVs [49] are gas-filled protein nanostructures that are originally located in the cells of some bacteria and archaea that regulate cell buoyancy in aqueous environments [50,51]. GVs are gene-encoded nanoscale probes composed of the primary structural protein, GvpA, the optional external scaffolding protein, GvpC, for structure reinforcement, and several secondary proteins that function as essential minor constituents or chaperones [52]. GVs consists of external hydrophilic and internal hydrophobic protein structures, which cause their interiors to form gas cavities filled with gas that is separated from the surrounding medium and realizes the simultaneous free exchange of internal and external gas [53]. In the biological world, photosynthetic bacteria use the contents of gas contained in vesicles to regulate buoyancy and accomplish their own floating behavior. The magnetic susceptibility of GVs is quite different from that of water, which can produce large contrasts in magnetic resonance imaging, even at sub-nanomolar concentrations. The gas cavities of the vesicles can scatter sound waves and produce ultrasonic contrasts. When the pressure on the air wall is greater than the threshold, the vesicles collapse; thus, background-free imaging can be achieved by acoustically modulated magnetic resonance imaging. The mechanical and surface characteristics of GvpC can be genetically modified by replacing the natural external GvpC with its recombinant variant, thus changing its magnetic susceptibility and collapse pressure, with the potential to obtain multichannel imaging. George et al. [54] showed that background-free imaging can be achieved by acoustic modulation MRI after injection of GVs into the striatum of mice. When using the same method, MRI contrast could not be obtained after injections of phosphate buffer without GVs. These results indicate that GVs are expected to become an MRI reporter gene for brain imaging.

Vasoactive peptides are another potential MRI reporter gene for use in brain imaging. Their expression can cause vasodilation and facilitate hemodynamic imaging. Designed probes based on vasodilating peptides can image brain regions [55] and can be used to detect important molecules in the brain, such as neurochemicals [56]. Its use provides the potential to examine a wide variety of molecular phenomena in the brain and other organs.

### 3.2. Brain Imaging of Reporter Genes with Radionuclide

The majority of radionuclide imaging studies in the brain are related to cell/gene therapy monitoring, as shown in Table 3 [57,58,59,60,61,62,63,64,65,66,67,68,69,70,71,72,73,74]. They must be able to address challenges such as penetrating the blood–brain barrier (BBB), imaging in regions of high endogenous gene expressions in the central nervous system (CNS), low specificity, and endogenous expressions of reporter genes in microglia [73]. Shimojo [57] used bacterial dihydrofolate reductase (ecDHFR) as a reporter gene and [^18^F]FE-TMP as an imaging probe, which functioned as a dual probe in both fluorescence and PET imaging to image the CNS system. As a result, PET could analyze mammalian brain circuits at the molecular level.

**Table 3:** Reporter genes and corresponding radiotracer in radionuclide brain imaging.

Class	Reporter Gene	Imaging Probe	Properties	Refs.
Enzyme	ecDHFR	[^18^F]FE-TMP	Allows PET analyses of mammalian brain circuits at the molecular level. TMP can be conjugated with fluorophores, while the radioactive analogs, [^11^c]TMP and [^18^F]TMP are compatible with PET.	[57]
	HSV1-TK	[^18^F]FHBG	Safely enables the longitudinal imaging of T cells stably transfected with a PET reporter gene in patients. Allows noninvasive monitoring of cell fate in cell therapy. Does not cross BBB.	[58,59,60]
	HSV1-TK	[^18^F]FHBT	Shows no statistically significant improvement of BBB permeability compared with [^18^F]FHBG.	[61]
	HSV1-TK	[^18^F]FIAU, [^18^F]FEAU	Potential PET imaging agents for suicide gene expression.	[62]
	HSV1-TK	[^124^I]FIAU	FIAU does not penetrate the intact BBB significantly.	[63]
	HSV1716	[^131^I]FIAU	SPECT in patients, intratumoral injection.	[64]
	HSV1-TK	[^131^I]FIAU	SPECT in rat. Local injection of stem cells is needed.	[65]
	HSV1-TK	[^76^Br]FBAU	Shows intracranial tumors.	[66]
Receptor	D2R	[^11^C]NMSP	Can assess the neural stem-cell-induced D2R expression in rat model.	[68]
	D2R80A	[^18^F]fallypride	A potent reporter to detect hMSCs (human mesenchymal stem cells) by PET in vivo.	[69,70]
	hCB(2)	[^11^C]GW405833	Dual-modality imaging viral vectors encoding hCB(2)(D80N) reporter system has potential clinical use as a PET reporter in the intact brain.	[71]
Transporter	hNIS	99mTc	SPECT in rat, for neural stem-cell tracing.	[72]
	Pyruvate kinase M2	[^18^F]DASA-23	Applicable in all areas of the CNS of mice without breaking the blood–brain barrier.	[73]
	DMT1	[^52^Mn^2+^]	Dual-modality PET/MR tracking of transplanted stem cells in the central nervous system.	[74]

ecDHFR, bacterial dihydrofolate reductase; D2R, the dopamine type 2 receptor; [^11^C]NMSP, [^11^C]N-methylspiperone; D2R80A, a mutant of the dopamine type 2 receptor; hCB(2), human type 2 cannabinoid receptor; [^11^C]-GW405833, [^11^C]-labeled CB(2) ligand; DMT1, divalent metal transporter 1; FIAU, fluoro-5-iodo-1-beta-d-arabinofuranosyluracil; FEAU, fluoro-5-ethyl-1-beta-d-arabinofuranosyluracil; FLT, fluoro-3′deoxy-3′-l-fluorothymidine; [^52^Mn^2+^], Mn based PET contrast agents.

SPECT and PET are useful in neuroscience research, especially in studies of neurodegeneration and neuro-oncology [64,75]. Stem = cell therapy offers new strategies for treating neurological diseases, such as Alzheimer’s disease, Parkinson’s disease, Huntington’s disease, and multiple sclerosis caused by the loss of different types of neurons and glial cells in the brain. SPECT [65,72] and PET [68,69,73] can trace and evaluate the function of stem cells in the nervous system [76]. Multimodality imaging using several reporter genes used dual [77,78] or triple [79] fusion reporter vectors to enable high-sensitivity detection of cells in living animals. A dual-membrane protein positron and gamma-imaging reporter system using sodium iodide symporter and mutant dopamine D-2 receptor transgenes was developed for brain tumor detection.

HSV1-TK using the imaging probe, FHBG, has been used in glioma treatments to monitor chimeric antigen receptor (CAR) T-cell biodistributions and proliferation [58,59]. A study of the imaging probe, FIAU, in patients showed that FIAU cannot penetrate an intact BBB [63,64]. After gene therapy, substantial levels of FIAU may be detected within areas of BBB disruption; hence, clinically relevant levels of HSV-1-tk gene expression in brain tumors can be detected [63]. FBAU [66] is another promising imaging probe that has been studied in glioma imaging based on a mouse model.

In addition to glioma, bone marrow stem cells (BMSCs) used in experimental middle cerebral artery occlusion (MCAO) rat models have been imaged with a reporter gene–probe system [65], consisting of the HSV1-tk and [^131^I]FIAU pair. BMSCs were introduced into MCAO rat models via local injections into the brain or via injections into the lateral ventricle, carotid artery, or tail vein. The quantity of injected dose per gram in infarcted brain tissue in rats receiving injections into the brain was significantly higher than that in rats receiving injections elsewhere. No differences were seen in the other cell transplantation groups. SPECT imaging with [^131^I]FIAU 24 h after injection provides peak target-to-nontarget count ratios. Neural stem cells have also been traced by SPECT [72]. The human sodium iodide symporter (hNIS) has been used as a reporter gene to track neural stem cells after transplantation in the brains of rats by using SPECT/CT imaging with technetium-99m to indicate the effectiveness and lack of interference with neural stem cell functioning. Dopamine type 2 receptor (D2R) and its mutant (D2R80A) have been used for neural stem cell tracing in the central nervous system. [^11^C]*N*-methylspiperone microPET has been proven useful in imaging neural stem-cell-induced D2R expressions in a rat model of traumatic brain injury [68]. It has also been proven in athymic rats that D2R80A is an effective reporter gene for human mesenchymal stem-cell detection in vivo [69]. In another study with mice and cats, a separate adeno-associated virus type 1 vector with identical gene expression control elements was co-injected with the D2R80A vector. This dual-vector approach allows the D2R80A gene to be used with any therapeutic gene and to be injected into a single site for monitoring [70].

The BBB penetration ability of imaging probes hampers the usage of reporter gene imaging. FHBT was studied to improve BBB permeability [61], but there were no significant improvements compared with the traditional probe, FHBG. It was demonstrated that the novel scaffold proposed in this study supports the development of a new imaging probe with better BBB permeability for HSV1-tk and its mutant in the future. This imaging probe combined with reporter genes other than HSV1-TK provides a better solution for crossing the intact BBB. The human-type 2 cannabinoid receptor (hCB(2)) related ligand, [^11^C]GW405833, for example, is readily distributed across the BBB. hCB(2)(D80N) was locoregionally overexpressed in the rat striatum by stereotactic injections of lentiviral and adeno-associated viral vectors. Kinetic PET revealed specific and reversible CB(2) binding of [^11^C]GW405833 in the transduced rat striatum. The hCB(2) expressions were followed for 9 months, which demonstrates the potential future clinical use of CB(2) as a PET reporter in the intact brain [71]. In another study [73], the PKM2 reporter gene was delivered to the brains of mice by adeno-associated virus (AAV9) via stereotactic injection. PET imaging at 8 weeks post AAV delivery showed that the AAV-injected mice had increases in [^18^F]DASA-23 brain uptake in the transduced sites. PKM2 can be used in the central nervous system to monitor gene and cell therapy without breaking the BBB.

**Figure 6:**
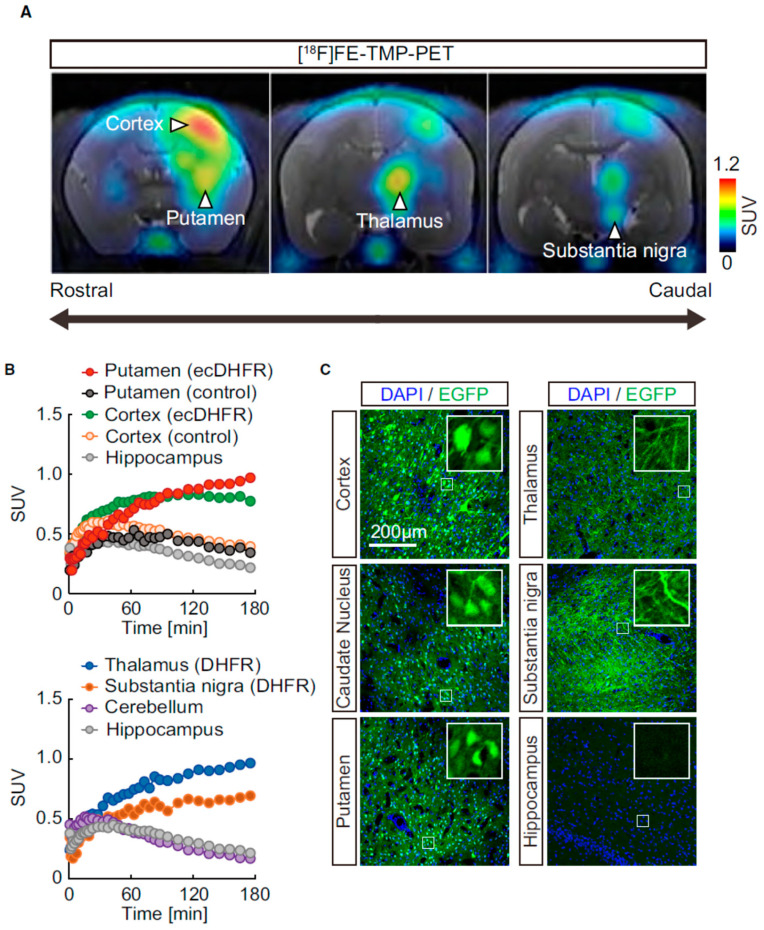
PET imaging of ecDHFR/TMP reporters in primate brain. (**A**) Representative coronal PET images generated by averaging dynamic scan data at 60–180 min after i.v. injection of [^18^F]FE-TMP. Note that reporter molecules were densely distributed in thalamus and substantia nigra pars compacta, which are connected to the neocortex and putamen via direct neuronal tract, respectively. PET images are overlaid with an individual MRI. Scale bar represents the SUV. (**B**) Time–radioactivity curves for [^18^F]FE-TMP in the putamen and neocortex carrying ecDHFR-EGFP or control AAV, and in the hippocampus are displayed in the upper panel. Curves in the thalamus, substantia nigra, and cerebellum along with hippocampus data are also shown in the lower panel. (**C**) Postmortem analysis of ecDHFR-EGFP expression in brain slices of different part with high-magnification image frames in inserts. Reproduced with permission from the authors (2021) [57].

Shimojo et al. [57] used bacterial dihydrofolate reductase, ecDHFR, and its unique antagonist, TMP, to visualize the neuronal circuit activities elicited by chemogenetic manipulation in the mouse hippocampus. In addition to mice, a 3.4 year old common marmoset underwent PET scans 45 days after AAV injection, when ecDHFR-EGFP was expressed in the brain. The PET results and postmortem fluorescence images are shown in Figure 6.

Biological variables are the byproducts of reporter gene expression PET imaging. Xu et al. [80] described a method that minimizes both the design and variability of vector delivery vehicles for alternative PET reporter genes (PRG) and the biological variability of the in vivo target when comparing the efficacy, sensitivity, and specificity of alternative PET reporter gene/PET reporter probe (PRP) combinations for in vivo PRG imaging. The Xu group described the process of comparing the standard HSVsr39TK/[^18^F]FHBG PRG/PRP reporter system to four other PRGs, which were all coupled with the same PRP, ^18^F-l-FMAU.

## 4. Opportunities and Challenges

### 4.1. About the Modalities

Four clinical imaging modalities have the potential for conducting reporter gene imaging. MRI is the most common modality in brain detection and study. The limitations of MRI are the sensitivity of available imaging agents, the cost, and the imaging time. PET and SPECT have been used in gene and cell therapy because of their high sensitivity. SPECT needs strict collimation to maintain resolution, which may cause an increase in radioisotope dose. PET is more sensitive and has higher resolution than SPECT. SPECT and PET are often combined with CT for co-registered anatomical and functional imaging. The reporter genes for ultrasound, namely, the acoustic reporter gene (ARG), are exclusively GVs. Studies in prokaryotic cells and mammalian cells have been carried out. Traditionally, imaging with probes that directly interact with target cells or molecules is called direct imaging. Imaging with probes that react to surrogate components that are related to the target cells or molecules is called indirect imaging. Imaging based on reporter genes is indirect. In existing brain studies, GVs can be used in brain imaging as probes but are not produced by target cells through transgenic technology. Further studies are needed to indirectly correlate GVs with target cells or molecules. The future of GVs as reporter genes or imaging contrast or therapy methods is very promising.

### 4.2. MRI

The high spatial resolution of MRI leads to its wide use in brain imaging research. The use of targeted contrast agents [81] has further improved its signal-to-noise ratio, and reporter gene technology has further improved the sensitivity and specificity of imaging. Some MR reporter genes (e.g., β-galactosidase) need to be used together with contrast agents to be detected, while some MR reporter genes (e.g., ferritin) do not need to add exogenous contrast agents, which has unique advantages compared with targeted contrast agents. On the one hand, the use of only reporter genes avoids the biological barriers that need to be overcome by injecting contrast media and the clearance barriers that contrast agents retain in the blood and tissue. Moreover, these reporter genes are generally not diluted with cell division-like targeted contrast agents (e.g., SPIO [82,83]), which can achieve more long-term and effective MRI monitoring [84,85]. On the other hand, reporter genes are generally expressed only in living cells, and they generally do not report both living and dead cells like imaging probes (e.g., the perfluorocarbon tracer), resulting in false-positive results [86,87,88].

Reporter gene-based MRI has great potential for use in brain imaging applications, but there are still many limitations to be overcome. Low sensitivity is a major limitation of MRI, while reporter genes based on CEST need high expression levels to achieve observable contrasts, which greatly limits the sensitivity of CEST. Reporter genes based on metal proteins or metal ion transporters may be hindered by the bioavailability or toxicity of metal ions. Therefore, there is a great demand for new MRI reporter genes that do not require metals and can be detected at low expression levels. The emergence of the aquaporin reporter gene improves these problems to some extent, but a potential limitation of aquaporin is its negative contrast enhancement.

In recent years, with the rapid development of MRI reporter genes, many innovative studies have been conducted. An interesting research direction is to integrate several types of reporter genes and combine the advantages of multiple imaging methods to achieve multimodal imaging. The search for new MRI reporter molecules is also an important research direction. In recent years, some studies have suggested that additional new molecules can be used as MRI reporter genes. For example, gas vesicles may become unique and powerful reporter genes in background-free MRI [54]. The ability to map several transgenes expressed at the same time by MRI will further enrich the gene transform palette and add additional “colors”, thus expanding the “multicolor” imaging toolbox to image previously inaccessible deep tissues [42]. However, most current research is currently in the animal experiment stage, and there is still a long way to go before they can be applied in clinics.

### 4.3. Radionuclide Imaging

Radionuclide brain imaging has been used in molecular imaging for tumor detection and therapy and has even exhibited the possibility of brain function studies. The SPECT-based reporter gene technique has been studied in patients [64]. The progress of gene expression, posttranscriptional events, and brain circuit function can be noninvasively visualized by repetitively using reporter genes. Most of the paired imaging probes of the studied reporter genes are already clinically approved, which causes reporter gene radionuclide imaging to be close to clinical usage. Cell tracking studies have experienced great improvements. [^18^F]FHBG imaging was safe in tracking HSV1-tk reporter gene expressions present in chimeric antigen receptor-engineered cytotoxic T lymphocytes and enabled the longitudinal imaging of T cells that were stably transfected with a PET reporter gene in patients [58].

The difficulty when choosing a reporter is that host-compatible reporters have no immune rejection risks, while their endogenous expressions may interfere with the real signal. Radionuclide imaging cannot be used in long-term studies due to decay of the imaging probe. The reporter gene will be expressed by the undiluted target cells for the lifetime of the cells and even for expanding cells. Until the design of viral tools for noninvasive gene delivery can apply these reporters to biomedical radionuclide imaging of human brains [89], researchers must face safety, cost-effectiveness, and ethics challenges.

HSV1-tk and its mutant are the most popular reporter genes for PET imaging. The inability to pass through the blood–brain barrier makes it difficult for them to be used in brain studies [68]. Imaging probes (e.g., PET tracers and reporter probes) that can cross the BBB have been developed [61,71,73]. A central nervous system multiparameter PET optimization (CNS PET MPO) algorithm was developed by a group from Pfizer that provides predictions of the required physicochemical properties of clinically useful CNS PET agents [61,90]. Reporter gene PET imaging is combined with other modalities, such as MRI [74] and optical imaging [71].

### 4.4. Pharmacodynamic and Pharmacokinetic Assessment

A wide spectrum of therapeutic viruses, genes, and cells have been developed in recent years for personalized treatment of cancers. Despite of many undergoing clinical trials and some now licensed for clinical use, the safety, accuracy, and efficiency of these therapies remain big concerns [91,92]. In vivo imaging tools are in urgent need for the longitudinal monitoring of the biological distribution and fate of these exogenous cells, genes, and viruses, as well as an evaluation of the therapeutic response and promotion of their clinical translation [91,93]. As one of the most promising approaches, reporter genes feature a high level of cellular specificity, without signal dilution, such as cellular proliferation or tumor metastasis. Among all the modalities, reporter genes expressing PET tracers have progressed quickly and are considered most valuable for clinical translation. Notably, the combination of PET and MRI reporter genes exhibits translational potential with superiority in both specificity and localization [94].

However, in practice, multiple factors need to be taken into accounts, including the background signal contaminations, the favorable dosage over radiation exposure, and the limited or no biological effect deriving from expression of the transgene. Additionally, the potential for nonhuman reporter genes to be immunogenic must also be taken into consideration when addressing ideal system design, since this may affect functionality and survival of transduced cells once injected into humans [2,14].

In addition to the performance improvement of reporter gene and imaging probe, the clinical usage of reporter gene imaging faces some major problems such as the stable transfer of reporter gene safely and efficient delivery of reporter gene into primary cells that cannot be cultured ex vivo for long period. Early collaborations between molecular imagers and cell gene therapists may solve these problems, which would afford a parallel approach starting with pre-clinical studies so that reporter gene imaging can be incorporated into the cell or gene therapy. Members of the molecular–genetic imaging industry such as CellSight Technologies have bridged the gap between reporter gene technologies and clinical trials [2,91].

### 4.5. Neuron Imaging with Reporter Gene

Reporter gene imaging for neuron imaging is a new rising direction with only a few studies reported. Wang’s group imaged astrocytes in the whole brain with engineered AAVs and DWI in vivo [39]. They also use ferritin-encoding trans-synaptic [32] and adeno-associated virus [33] to detect neural connections ex vivo and longitudinal neural connections in vivo, respectively. Shimojo et al. [57] used ecDHFR and TMP to visualize the neuronal circuit activities elicited by chemo-genetic manipulation in the mouse hippocampus. These studies provide substitutes for genetically encoded fluorescent probes, enabling in vivo gene targeted neuronal activity observation to be carried out in real time in larger animals. Hence, more mechanisms of neuron activity will be discovered in nonhuman primates or even humans in the future.

## 5. Conclusions

Reporter genes for use with clinical imaging modalities have greatly improved in recent years. For brain imaging studies, in addition to tumor detection and therapy, brain function-related studies cut a striking figure. Advances in gene techniques will bring great progress in brain imaging. The development of molecular functional MRI tools for animal research is accelerating, but the maximum benefits of this technology will eventually be achieved in human subjects. The abovementioned reporter gene technologies should be used and should not be limited to proof-of-concept experiments. Only in this way will it be possible to recognize the practical limitations of the emerging reporting mechanisms and identify important ways to improve them.

## Figures and Tables

**Table 1:** Pros and cons of reporter gene brain imaging techniques.

	MRI	PET	SPECT	US	Optical Imaging
Penetration depth	Limitless	Limitless	Limitless	>10 cm, limited in hard and air-containing tissue	250–500 μm
Field of view	Whole body	Whole body	Whole body	Whole organ	1–2 mm^2^
Spatial resolution	100–1000 μm	4–7 mm	1 cm	50–500 μm	Poor at greater depths
Temporal resolution	100–1000 ms	s	min	1–100 ms	Good
Detection capability	μM	pM-fM	pM	pM	nM
Imaging time	min–h	min–h	min–h	s–min	ms–min
Ionization radiation	No	Yes	Yes	No	No
Clinical utility	Yes	Yes	Yes	Yes	Limited
Sensitivity	poor	Excellent	Excellent	excellent	Excellent
Information	Anatomical, physiological, molecular	Physiological, molecular	Physiological, molecular	Anatomical physiological	Physiological, molecular

## Abbreviations

Abbreviations	Full name
MRI	Magnetic resonance imaging
TfR	Transferrin receptor
AQP1	Aquaporin 1
CEST	Chemical exchange saturation transfer
LRP	Lysine-rich protein
FTH	Ferritin heavy chain
FerrH	Ferritin heavy chain
DWI	Diffusion-weighted imaging
ADC	Apparent diffusion coefficient
EGFP	Enhanced green fluorescent protein
T2WI	T2-weighted image
FI	Fluorescence imaging
MSCs	Mesenchymal stem cells
SWI	Susceptibility-weighted imaging
BMSCs	Bone marrow mesenchymal stromal cells
IFNβ	Interferon-β
iRFP	Near-infrared fluorescent protein
rd LRP	Redesigned LRP reporter
dNKs	Deoxyribonucleoside kinases
VSV	Vesicular stomatitis virus
rVSV	Recombinant VSV
SC	Somatosensory cortex
AAV	Adeno-associated virus
CPU	Caudate putamen
Ctx	Cerebral cortex
BLA	Basolateral amygdala
Ins	Insular cortex
Tha	Thalamus
HIP	Hippocampus
GVs	Gas vesicles
PET	Positron emission tomography
SPECT	Single-photon emission computed tomography
US	Ultrasound
HSV1-tk	Herpes simplex virus-1 thymine kinase
hΔtk2	Human Δ-mitochondrial thymine kinase type 2
hSSTR2	Human somatostatin receptor type 2
D2R	Dopamine type 2 receptor
h NIS	Human sodium–iodide symporter
h NET	Human norepinephrine transporter
ecDHFR	Bacterial dihydrofolate reductase
[^11^C]NMSP	[^11^C]*N*-methylspiperone
D2R80A	Mutant of the dopamine type 2 receptor
hCB(2)	Human type 2 cannabinoid receptor
[^11^C]-GW405833	[^11^C]-labeled CB(2) ligand
DMT1	Divalent metal transporter 1
FIAU	Fluoro-5-iodo-1-beta-d-arabinofuranosyluracil
FEAU	Fluoro-5-ethyl-1-beta-d-arabinofuranosyluracil
FLT	Fluoro-3′deoxy-3′-l-fluorothymidine
[^52^Mn^2+^]	Mn-based PET contrast agents
CAR	Chimeric antigen receptor
BMSCs	Bone marrow stem cells
MCAO	Middle cerebral artery occlusion
PRG	PET reporter gene
PRP	PET reporter probe
ARG	Acoustic reporter gene
CNS PET MPO	Central nervous system multiparameter PET optimization
